# Predictive Value of Laboratory Assays Toward Autoimmune Diseases

**DOI:** 10.1002/jcla.70106

**Published:** 2025-10-23

**Authors:** Kellen J. Begeman, Mark J. White, Elizabeth S. Moore, Heidi H. Ewen

**Affiliations:** ^1^ University of Indianapolis Indianapolis Indiana USA; ^2^ University of Indianapolis, Indianapolis, INLabcorp, Burlington, NC USA

**Keywords:** anti‐nuclear antibody, autoimmune disease, blood urea nitrogen, complement c3, complement c4, globulin, monocyte‐to‐lymphocyte ratio, neutrophil‐to‐lymphocyte ratio, pH, urine protein

## Abstract

**Objective:**

With an increasing number of unmet medical needs for autoimmune diseases (AD) in the United States, it is important to assess the value of laboratory assays in diagnosis. Healthcare providers must understand general and specific laboratory results that can lead to an efficient diagnosis.

**Methods:**

A nonexperimental study using a retrospective design and data collected between 2018 and 2021 was completed to explore whether the presence of positive anti‐ENA and anti‐dsDNA antibodies in individuals with an autoimmune disorder (anti‐ENA, *N* = 1495; anti‐dsDNA, *N* = 1261) can be predicted by their demographics and selected general laboratory test results.

**Results:**

For predicting anti‐ENA, multiple logistic regression analysis *χ* ^2^(1) = 237.62, *p* < 0.001 indicated antinuclear antibody (ANA), complement C3, globulin, monocyte‐to‐lymphocyte ratio (MLR), and blood urea nitrogen (BUN) were statistically associated with the probability of a positive anti‐ENA result. The regression analysis had a sensitivity of 98.6% and a specificity measure of 16.1%. Regarding anti‐dsDNA, multiple logistic regression analysis *χ* ^2^(1) = 388.04, *p* < 0.001, indicated that complement C3, complement C4, pH, urine protein, and neutrophil‐to‐lymphocyte ratio (NLR) were statistically associated with the probability of a positive anti‐dsDNA result. The regression analysis had a sensitivity of 77.7% and a specificity measure of 64.5%.

**Conclusion:**

In diagnosing an AD, ANA, complement C3, globulin, MLR, and BUN are important factors in patients with a positive anti‐ENA. In determining Systemic Lupus Erythematosus diagnosis, complement C3, complement C4, pH, urine protein, and NLR are important factors in patients with a positive anti‐dsDNA.

## Graphical abstract

With an increasing number of unmet medical needs for autoimmune diseases (AD) in the United States, it is important to assess the value of laboratory assays in diagnosis. Healthcare providers must understand general and specific laboratory results that can lead to an efficient diagnosis. In diagnosing an AD, antinuclear antibody, complement C3, globulin, monocyte‐to‐lymphocyte ratio, and blood urea nitrogen are important factors in patients with a positive anti‐ENA. In determining Systemic Lupus Erythematosus diagnosis, complement C3, complement C4, pH, urine protein, and neutrophil‐to‐lymphocyte ratio are important factors in patients with a positive anti‐dsDNA.

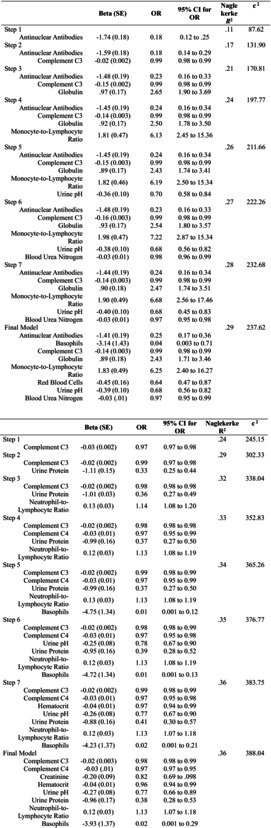

Worldwide, 5%–10% of individuals suffer from autoimmune diseases (ADs), with 15 to 20 million cases in the United States, culminating as the third leading cause of illness and mortality [1]. Autoimmunity constitutes elements of an organism's immune system attacking its own tissue or cells [2]. The corresponding formed antibodies, known as autoantibodies, contribute to the diseases known as ADs [2].

ADs currently reflect a significant unmet medical need and clinical problem due to their associated healthcare cost, chronic status, and prevalence in younger populations [3]. Therapy for ADs is typically continuous and lifelong, presenting an increased risk of complications in malignancies and infections [3]. A major contributor to healthcare costs is not only medications for the management of ADs but also the cost of laboratory screening and diagnostic testing [4]. The initial clinical investigation remains expensive, as ADs are notoriously difficult to confirm, requiring not only medical specialists but also increasingly more specific, complex, and costly laboratory analyses [3, 5].

Despite the expense, laboratory testing is extremely valuable for evaluating suspected ADs. Results have the potential to not only assist with diagnoses but also estimate disease severity and the tracking and prognosis of disease activity [6]. General laboratory testing can be conducted to begin probing the possibility of an AD. Health providers may review erythrosedimentation rate (ESR), comprehensive metabolic panel (CMP), c‐reactive protein (CRP), complete blood count (CBC), and urinalysis (UA, 7). Even more specific are the detections of the actual autoantibodies to confirm the preliminary diagnosis made from general laboratory results, and they have the most prognostic impact and value in monitoring treatment response [2]. Screening tests detect antinuclear antibodies (ANA), followed by anti‐double stranded DNA (anti‐dsDNA) antibodies and anti‐extractable nuclear antigen antibodies (anti‐ENA), which are generally highly specific for confirmation of a type of AD. Although the importance of the management of the disease is evident and, when evaluated correctly, can lead to prompt diagnosis and treatment, positive results can, unfortunately, complicate patient care [2]. Some autoantibodies are present in healthy populations, leading to false positives, which in turn can cause the administration of inappropriate and potentially damaging treatment [2, 7].

## 1 Research Problem

The diagnostic responsibility for a potential AD lies with the attending physician as it requires deciphering testing complexities. If discrepancies are acknowledged or results do not fit into the clinical context or physician expectations, the specimens can go through repeat analysis or testing using another methodology or assay with its own characteristics in terms of performance and accuracy [1]. Additionally, the risk of false positives increases as a physician requests further measurement of multiple antibodies [1]. False positivity is demonstrated as anti‐ENA detection sensitivity and specificity, respectively, according to the technique: enzyme‐linked immunosorbent assay (ELISA) (50.0%, 78.9%); double immunodiffusion (31.3%, 89.5%); hemagglutination tests (40.9%, 87.7%) [8].

Even with advancements in laboratory technology and diagnostic tools, the presence of an autoantibody does not singularly confirm AD diagnosis, nor does its absence exclude diagnosis. A physician's knowledge of each laboratory test's diagnostic power and usage of the most meaningful assays concerning ADs is essential in complementing physical examination [2]. Studies have assessed the value and utility of singular and multiple variables of laboratory results to assist in diagnosing ADs. However, no other studies have evaluated the predictive value of all the essential general and specific diagnostic laboratory tests available to confirm ADs.

## 2 Purpose Statement

The purpose of this study was to provide healthcare providers with the value and utility of laboratory analysis for diagnosing a suspected AD.

## 3 Research Question

The following research question was answered to meet the study's purpose. Is there a relationship between age, gender, CRP, CBC, UA, CMP, ANA, anti‐ENA, and anti‐dsDNA antibodies?

To answer the research question, the following objective was addressed: To determine the predictive value of age, gender, CRP, CBC, UA, CMP, and ANA test results for the presence of anti‐ENA and anti‐dsDNA antibodies using multiple logistic regression analysis (Appendix A).

## 4 Significance of the Study

ADs undoubtedly contribute significantly to healthcare costs and an affected individual's quality of life and longevity [1]. Complex disease demonstration in patients, coupled with the diagnostic limitations of laboratory testing, increases the potential for an incorrect diagnosis. Misdiagnosis can lead to inaccurate treatment, and with a heavy reliance on laboratory analysis, providers cannot afford to make mistakes in interpreting results [2]. With guidance on the true value and utility of each laboratory test concerning the diagnosis of an AD, physicians can feel more confident in their approach and treatment, patients can receive the correct and critical medical AD diagnosis, and the cost burden of unnecessary laboratory screening tests can be reduced.

## 5 Method

### 5.1 Study Type and Design

A nonexperimental study using a retrospective design was completed to explore whether the presence of positive anti‐ENA and anti‐dsDNA antibodies in individuals with an AD can be predicted by their demographics and selected general laboratory test results. The study took place from November 1, 2022, to December 6, 2023. Blood cell ratios were calculated by dividing the absolute cell count by the absolute cell count of the target cell lines, for example, absolute monocyte count/absolute lymphocyte count. All other ratios were provided within the retrospective laboratory results.

### 5.2 Study Population

The population from which the samples were drawn is individuals who had biological specimens obtained and tested between 2018 and 2021 by a diagnostic laboratory company located in the United States. Potential participants were identified from a database that consists of patients who received laboratory testing from a diagnostic laboratory. To be included in the study, the individual was screened for and had the following data points recorded: age, gender, CRP, CBC, UA, CMP, ANA to anti‐ENA, and anti‐dsDNA antibody results. Participants were excluded if they had a known co‐morbid underlying condition other than an AD based on the provided retrospective data. Participant treatments or therapies were not included in the data. The anti‐ENA population consisted of 1345 females and 150 males with a mean age of 53. The anti‐dsDNA population consisted of 1106 females and 155 males with a mean age of 52. Underlying autoimmune diseases were based on the associated dependent variable positivity (Table 1).

**TABLE 1:** Anti‐ENA positive sample: Sensitivity, specificity, associated disease(s).

ENA	Sample (*N*)	Sensitivity/specificity (%)	Associated disease(s)
Anti‐centromere B antibodies	100	94, 93	Scleroderma CREST variant
Antichromatin antibodies	230	99, 100	SLE
Anti‐Jo‐1	1	45, 95	Myositis
Antiribosomal P antibodies	4	99, 32	SLE
Antiscleroderma‐70 antibodies	57	45, 90	Scleroderma
RNP antibodies	412	98, 60	Mixed connective tissue disease (MCTD)
Sjogren's Anti‐SS‐A	383	97,50	Sjögren's syndrome, SLE
Sjogren's Anti‐SS‐B	74	85, 60	Sjögren's syndrome
Smith antibodies	60	50, 99	SLE

*Note:* Sensitivity and specificity percentages acquired based on ELISA methodology [9, 10, 11, 12].

### 5.3 Data

A waiver of informed consent and an exempt review was obtained from the University of Indianapolis Institutional Review Board, as illnesses are not considered terminal. Also, the research involves the collection and study of existing data from pathological and diagnostic specimens with numeric identifiers linked to subjects. Only retrospective data were collected, and there was no direct participant interaction.

All data were extracted from the Laboratory Information System (LIS) and stored on a secured external drive. Only the primary researcher (K.J.B.) had access to the data by accessing a computer system protected by a personal username and password, limiting data transfer from its original source.

### 5.4 Statistical Analysis

Descriptive statistical analyses were used to determine mean values and standard deviations for all quantitative laboratory parameters and demographics (Tables 2 and 3). Multiple logistic regression analyses were used to determine if laboratory test results and demographic information could predict the dependent variables, anti‐ENA screen, and anti‐dsDNA screen. Any correlations between independent and dependent variables less than 0.001 were selected as possible predictors and entered into the regression model. Assumptions for multiple logistic regression were tested and interpreted, including a sample representative of the population, dependent variables measured on a nominal dichotomous scale with infinite probability values between 0 and 1, independent predictor variables of nominal, ordinal, interval, or ratio scale, no multicollinearity, sufficient sample size, linearity of the continuous predictor variable, and log odds of the dependent variable. The Nagelkerke *R* ^2^ was used to determine how much the independent variables predicted the dependent variable, with an increase demonstrating goodness of fit. For the inclusion of variables, we ensured that the model improved upon each addition.

**TABLE 2:** Descriptive statistics anti‐ENA.

ENA variables	Mean	Standard deviation
Age (years)	52.62	16.53
Albumin globulin (AG) ratio	1.57	0.52
Albumin	4.30	0.62
Alkaline Phosphatase	94.88	54.46
Alanine aminotransferase (ALT)	36.28	51.59
Aspartate aminotransferase (AST)	38.07	59.68
Bilirubin Serum	0.51	0.33
Blood urea nitrogen (BUN)	18.49	11.29
BUN creatinine ratio	20.27	8.27
Calcium	9.54	0.69
Chloride	103.94	4.22
Globulin	3.28	0.71
Potassium	4.57	0.55
Protein	7.46	0.83
Sodium	141.46	3.63
C‐reactive protein (CRP)	12.37	23.30
Eosinophil absolute	0.21	0.23
Hematocrit	38.01	5.69
Lymphocytes absolute	1.82	1.37
Monocytes absolute	0.63	0.25
Neutrophils absolute	5.13	2.94
Basophils absolute	0.03	0.05
Platelets	297.35	109.74
Red blood cells (RBCs)	4.40	0.76
Red blood cell width (RDW)	14.95	2.18
White blood cells (WBC)	7.37	3.73
Eosinophil‐to‐lymphocyte ratio (ELR)	0.16	0.22
Monocyte‐to‐lymphocyte ratio (MLR)	0.52	0.67
Neutrophil‐to‐lymphocyte ratio (NLR)	4.55	6.85
Platelet‐to‐lymphocyte ratio (PLR)	253.89	338.11
Creatinine	1.01	0.83
pH	6.47	0.89
Specific gravity	1.02	0.01
Erythrosedimentation rate (ESR)	38.61	30.38
Complement C3	118.30	38.51
Complement C4	21.44	11.06

**TABLE 3:** Descriptive statistics anti‐dsDNA.

DSDNA variables	Mean	Standard deviation
Age (years)	51.99	16.92
Albumin globulin (AG) Ratio	1.60	0.51
Albumin	4.28	0.65
Alkaline phosphatase	94.65	53.68
Alanine aminotransferase (ALT)	37.75	61.79
Aspartate aminotransferase (AST)	38.21	63.01
Bilirubin serum	0.53	0.34
Blood urea nitrogen (BUN)	19.17	11.78
BUN creatinine ratio	20.69	8.13
Calcium	9.51	0.72
Chloride	104.01	4.06
Globulin	3.24	0.69
Potassium	4.58	0.55
Protein	7.38	0.88
Sodium	141.53	3.59
C‐reactive protein (CRP)	12.18	21.85
Eosinophil absolute	0.22	0.20
Hematocrit	38.43	5.86
Lymphocytes absolute	1.91	1.15
Monocytes absolute	0.62	0.25
Neutrophils absolute	5.30	2.98
Basophils absolute	0.04	0.05
Platelets	299.06	109.54
Red blood cells (RBCs)	4.47	0.74
Red blood cell width (RDW)	15.03	2.25
White blood cells (WBC)	7.65	3.56
Eosinophil‐to‐lymphocyte ratio (ELR)	0.16	0.20
Monocyte‐to‐lymphocyte ratio (MLR)	0.50	0.72
Neutrophil‐to‐lymphocyte ratio (NLR)	4.71	7.62
Platelet‐to‐lymphocyte ratio (PLR)	247.79	350.37
Creatinine	1.04	0.83
pH	6.57	0.91
Specific gravity	1.02	0.01
Erythrosedimentation rate (ESR)	37.44	29.98
Complement C3	116.98	39.37
Complement C4	21.22	11.01

Data were analyzed using IBM SPSS Statistics for Windows, Version 24.0 (IBM Corp., Armonk, NY). The assumption that the models reasonably fit the linearity assumption was based on chi‐square significance. A significance level of less than 0.001 was considered statistically significant.

## 6 Results

### 6.1 Anti‐ENA

Multiple logistic regression analysis using stepwise variable entry indicated that ANA, complement C3, globulin, monocyte‐to‐lymphocyte ratio (MLR), and blood urea nitrogen (BUN) were statistically associated with the probability of a positive anti‐ENA result (Table 4). The final model achieved an acceptable fit, *χ* ^2^(1) = 237.62, *p* < 0.001. From the sample of 1495 patients, 1321 patients (88%) were observed to have a positive anti‐ENA. The regression analysis correctly predicted 1302 of the 1321 participants with positive anti‐ENA for a sensitivity of 98.60%. Also, in this sample, 174 patients did not have a positive anti‐ENA. The logistic regression analysis correctly classified 28 of the 174 participants as not having a positive anti‐ENA with a specificity measure of 16.10%. In the overall classification predictive model, 89% of the participants were correctly classified as anti‐ENA positive (true positive) or anti‐ENA negative (true negative).

**TABLE 4:** Multiple logistic regression using stepwise variable entry to predict anti‐ENA.

	Beta (SE)	OR	95% CI for OR	Naglekerke *R* ^2^	*c* ^2^
Step 1				0.11	87.62
Antinuclear antibodies	−1.74 (0.18)	0.18	0.12 to 0.25		
Step 2				0.17	131.90
Antinuclear antibodies	−1.59 (0.18)	0.18	0.14 to 0.29		
Complement C3	−0.02 (0.002)	0.99	0.98 to 0.99		
Step 3				0.21	170.81
Antinuclear antibodies	−1.48 (0.19)	0.23	0.16 to 0.33		
Complement C3	−0.15 (0.002)	0.99	0.98 to 0.99		
Globulin	0.97 (0.17)	2.65	1.90 to 3.69		
Step 4				0.24	197.77
Antinuclear antibodies	−1.45 (0.19)	0.24	0.16 to 0.34		
Complement C3	−0.14 (0.003)	0.99	0.98 to 0.99		
Globulin	0.92 (0.17)	2.50	1.78 to 3.50		
Monocyte‐to‐lymphocyte ratio	1.81 (0.47)	6.13	2.45 to 15.36		
Step 5				0.26	211.66
Antinuclear antibodies	−1.45 (0.19)	0.24	0.16 to 0.34		
Complement C3	−0.15 (0.003)	0.99	0.98 to 0.99		
Globulin	0.89 (0.17)	2.43	1.74 to 3.41		
Monocyte‐to‐lymphocyte ratio	1.82 (0.46)	6.19	2.50 to 15.34		
Urine pH	−0.36 (0.10)	0.70	0.58 to 0.84		
Step 6				0.27	222.26
Antinuclear antibodies	−1.48 (0.19)	0.23	0.16 to 0.33		
Complement C3	−0.16 (0.003)	0.99	0.98 to 0.99		
Globulin	0.93 (0.17)	2.54	1.80 to 3.57		
Monocyte‐to‐lymphocyte ratio	1.98 (0.47)	7.22	2.87 to 15.34		
Urine pH	−0.38 (0.10)	0.68	0.56 to 0.82		
Blood urea nitrogen	−0.03 (0.01)	0.98	0.96 to 0.99		
Step 7				0.28	232.68
Antinuclear antibodies	−1.44 (0.19)	0.24	0.16 to 0.34		
Complement C3	−0.14 (0.003)	0.99	0.98 to 0.99		
Globulin	0.90 (0.18)	2.47	1.74 to 3.51		
Monocyte‐to‐lymphocyte ratio	1.90 (0.49)	6.68	2.56 to 17.46		
Urine pH	−0.40 (0.10)	0.68	0.45 to 0.83		
Blood urea nitrogen	−0.03 (0.01)	0.97	0.95 to 0.98		
Final model				0.29	237.62
Antinuclear antibodies	−1.41 (0.19)	0.25	0.17 to 0.36		
Basophils	−3.14 (1.43)	0.04	0.003 to 0.71		
Complement C3	−0.14 (0.003)	0.99	0.98 to 0.99		
Globulin	0.89 (0.18)	2.43	1.71 to 3.46		
Monocyte‐to‐lymphocyte ratio	1.83 (0.49)	6.25	2.40 to 16.27		
Red blood cells	−0.45 (0.16)	0.64	0.47 to 0.87		
Urine pH	−0.39 (0.10)	0.68	0.56 to 0.82		
Blood urea nitrogen	−0.03 (0.01)	0.97	0.95 to 0.99		

The logistic regression model showed statistically significant probability prediction and effectively classified patients with anti‐ENA positive results and patients without anti‐ENA positive results. The final model had a Nagelkerke *R* ^2^ of 0.29 (coefficient of determination) in this logistic regression analysis, with an overall predictive improvement in each variable entry step. The model converged in 5 iterations (−2 Log 1075.401).

The final model demonstrated that a positive anti‐ENA result was best predicted by ANA, basophils, complement C3, globulin, MLR, red blood cells (RBCs), urine pH, and BUN. Holding all other predictor variables constant, it was found that the odds of a positive anti‐ENA occurring decreased by 75% for the absence of ANA. The odds of a positive anti‐ENA occurring decreased by 96% for a one‐unit decrease in basophils, decreased by 1% for a one‐unit decrease in complement C3, increased by 143% for a one‐unit increase in globulin, increased by 525% for a one‐unit increase in MLR, decreased by 36% for a one‐unit decrease in RBC count, decreased by 32% for a one‐unit decrease in urine pH, and decreased by 3% for a one‐unit decrease in BUN (BUN; Table 4). Significant predictor variables were included in Equation (1) for the determination of a positive anti‐ENA.

(1) $$\begin{array}{cc} \text{Logit}\left(p\right)= & 6.07+\left(-1.41\times \mathrm{ANA}\right)+\left(-0.01\times \text{Complement C3}\right)\\ & +\left(0.89\times \text{Globulin}\right)+\left(1.83\times \mathrm{MLR}\right)+\left(-0.03\times \mathrm{BUN}\right) \end{array}$$

### 6.2 Anti‐dsDNA

Multiple logistic regression analysis *χ* ^2^(1) = 388.04 (*p* < 0.001) using stepwise variable entry resulted in a final model that indicated that complement C3, complement C4, creatinine, hematocrit, urine pH, urine protein, neutrophil‐to‐lymphocyte ratio (NLR), and basophils were statistically associated with the probability of a positive anti‐dsDNA result (Table 5). From the sample of 1261 patients, 771 patients (61%) were observed to have a positive anti‐dsDNA. The regression analysis correctly predicted 599 of the 771 participants with positive anti‐dsDNA for a sensitivity of 77.7%. In this sample, 490 patients did not have a positive anti‐dsDNA. The final logistic regression model correctly classified 316 of the 490 participants as not having a positive anti‐dsDNA with a specificity measure of 64.5%. In the overall classification predictive model, 72.5% of the participants were correctly classified as anti‐dsDNA positive (true positives) or anti‐dsDNA negative (true negative).

**TABLE 5:** Multiple logistic regression using stepwise variable entry to predict anti‐dsDNA.

	Beta (SE)	OR	95% CI for OR	Naglekerke *R* ^2^	*c* ^2^
Step 1				0.24	245.15
Complement C3	−0.03 (0.002)	0.97	0.97 to 0.98		
Step 2				0.29	302.33
Complement C3	−0.02 (0.002)	0.99	0.97 to 0.98		
Urine protein	−1.11 (0.15)	0.33	0.25 to 0.44		
Step 3				0.32	338.04
Complement C3	−0.02 (0.002)	0.98	0.98 to 0.98		
Urine Protein	−1.01 (0.03)	0.36	0.27 to 0.49		
Neutrophil‐to‐lymphocyte ratio	0.13 (0.03)	1.14	1.08 to 1.20		
Step 4				0.33	352.83
Complement C3	−0.02 (0.002)	0.98	0.98 to 0.98		
Complement C4	−0.03 (0.01)	0.97	0.95 to 0.99		
Urine protein	−0.99 (0.16)	0.37	0.27 to 0.50		
Neutrophil‐to‐lymphocyte ratio	0.12 (0.03)	1.13	1.08 to 1.19		
Step 5				0.34	365.26
Complement C3	−0.02 (0.002)	0.99	0.98 to 0.99		
Complement C4	−0.03 (0.01)	0.97	0.95 to 0.99		
Urine protein	−0.99 (0.16)	0.37	0.27 to 0.50		
Neutrophil‐to‐lymphocyte ratio	0.13 (0.03)	1.13	1.08 to 1.19		
Basophils	−4.75 (1.34)	0.01	0.001 to 0.12		
Step 6				0.35	376.77
Complement C3	−0.02 (0.002)	0.98	0.98 to 0.99		
Complement C4	−0.03 (0.01)	0.97	0.95 to 0.98		
Urine pH	−0.25 (0.08)	0.78	0.67 to 0.90		
Urine protein	−0.95 (0.16)	0.39	0.28 to 0.52		
Neutrophil‐to‐lymphocyte ratio	0.12 (0.03)	1.13	1.08 to 1.19		
Basophils	−4.72 (1.34)	0.01	0.001 to 0.13		
Step 7				0.36	383.75
Complement C3	−0.02 (0.002)	0.99	0.98 to 0.99		
Complement C4	−0.03 (0.01)	0.97	0.95 to 0.98		
Hematocrit	−0.04 (0.01)	0.97	0.94 to 0.99		
Urine pH	−0.26 (0.08)	0.77	0.67 to 0.90		
Urine protein	−0.88 (0.16)	0.41	0.30 to 0.57		
Neutrophil‐to‐lymphocyte ratio	0.12 (0.03)	1.13	1.07 to 1.18		
Basophils	−4.23 (1.37)	0.02	0.001 to 0.21		
Final model				0.36	388.04
Complement C3	−0.02 (0.003)	0.98	0.98 to 0.99		
Complement C4	−0.03 (0.01)	0.97	0.97 to 0.95		
Creatinine	−0.20 (0.09)	0.82	0.69 to 0.098		
Hematocrit	−0.04 (0.01)	0.96	0.94 to 0.99		
Urine pH	−0.27 (0.08)	0.77	0.66 to 0.89		
Urine protein	−0.96 (0.17)	0.38	0.28 to 0.53		
Neutrophil‐to‐lymphocyte ratio	0.12 (0.03)	1.13	1.07 to 1.18		
Basophils	−3.93 (1.37)	0.02	0.001 to 0.29		

The logistic regression model showed statistically significant probability prediction and effectively classified patients with anti‐dsDNA positive results and patients without anti‐dsDNA positive results. Nagelkerke *R* ^2^, the final model coefficient of determination, was 0.36 for this logistic regression analysis. Each step of the model showed overall improvement in the coefficient of determination and chi‐square model fitting indices. The model converged in 3 iterations (−2 Log 1684.971).

It was found that holding all other predictor variables constant, the odds of a positive anti‐dsDNA occurring decreased by 2% for a one‐unit decrease in complement C3, decreased by 3% for a one‐unit decrease in complement C4, decreased by 18% for a one‐unit decrease in creatinine, decreased by 4% for a one‐unit decrease in hematocrit, decreased by 23% for a one‐unit decrease in urine pH, decreased by 62% for the absence of urine protein, increased by 13% for a one‐unit increase in NLR, and decreased by 98% for a one‐unit decrease in basophils (Table 5). Significant predictor variables were included in Equation (2) for the determination of a positive anti‐dsDNA.

(2) $$\begin{array}{cc} \text{Logit}\left(p\right)= & 7.01+\left(-0.02\times \text{Complement C3}\right)+\left(-0.03\times \text{Complement C4}\right)\\ & +\left(-0.27\times \mathrm{pH}\right)+\left(-0.96\times \text{Urine Protein}\right)+\left(0.12\times \mathrm{NLR}\right) \end{array}$$

## 7 Discussion

The diagnosis of AD is notoriously difficult, requiring medical specialist examination and complex, costly laboratory tests [3, 5]. With the significant contribution to healthcare costs and detriment to an individual's quality of life and longevity, healthcare providers cannot afford to make mistakes in their diagnostic approach [3]. Laboratory analysis is valuable and useful for the proper diagnosis of suspected AD. For this reason, our study attempted to identify the association between laboratory assays related to ADs and their predictive value for better confidence in diagnosis and, ultimately, better patient outcomes. Through multiple logistic regression analyses, the relationship between laboratory analytes and demographic characteristics on the presence of anti‐dsDNA antibodies and anti‐ENA antibodies was studied.

### 7.1 Anti‐ENA

According to a multiple logistic regression model, ANA, complement C3, globulin, MLR, and BUN were significantly correlated to a positive anti‐ENA result. Although anti‐ENAs have strong clinical associations with several ADs, there is considerable overlap for the associated diseases. Anti‐ENAs still offer an efficient test procedure for the laboratory workup of patients with a suspected AD; therefore, for additional clarity, specific ENA positivity and associated disease(s) are outlined for our population (Table 1). Regarding overlap, for systemic lupus erythematosus (SLE), anti‐smith antibody (anti‐Sm) is highly specific, and anti‐ribonucleoprotein antibody (anti‐RNP) is also associated with the disease [13]. These anti‐ENAs were captured within this analysis, creating the potential for significant analytes to be associated with SLE. In particular, BUN is used to assess renal function by quantifying urea in the blood [14]. SLE commonly impacts renal function; therefore, the findings related to BUN may be attributable to SLE.

Another consideration for the overlap with SLE relates to ANA, as patients with the disease have demonstrated variable detection of ANA in their serum [15]. However, other studies have shown ANA as a hallmark of SLE among commercially available kits [16]. Our study demonstrated significance between ANAs and anti‐ENA, but it was not found significant toward anti‐dsDNA, a common marker for SLE, and ANAs, demonstrating the same variability acknowledged by previous researchers.

The first marker of significance within our model is ANA, observed in many patients with AD, and is one of the most common biomarkers of autoimmunity [17]. As autoantibodies are a distinctive hallmark used to classify autoimmune diseases, and ANA can detect them, our research aligns with their significance [18]. Our study demonstrated that the absence of an ANA significantly decreased the likelihood of having a positive anti‐ENA. Previous studies have shown that the ANA titer alone is useful in predicting anti‐ENA antibodies [19]. Within our results, a direct ANA, signifying a qualitative positive versus negative result, was significant in determining anti‐ENA presence. Additionally, titer and pattern are not necessarily needed to determine the presence of anti‐ENA, but a qualitative test result will suffice. This is important, as titration of an ANA, which produces a semiquantitative result, consumes laboratory and healthcare resources and the time of laboratory professionals and can create delays in the diagnostic process, which impacts the outcomes of AD patients.

Complement, which is activated in nearly all antibody‐mediated autoimmune diseases, is also present within our patient population [20]. Although it is an important component of the innate immune system, it also has a pathogenic role in many autoimmune systems, which can lead to tissue damage [21, 22]. Specifically related to the positive anti‐ENA disorder, primary Sjögren's syndrome (pSS), C3 hypocomplementemia has been established as a clinical feature and associated with extraglandular manifestations, suggesting clinical value within pSS patients [23]. Antiphospholipid syndrome, an anti‐ENA positive disease, is also associated with C3 deficiency [22]. Our study demonstrated that with decreases in C3, the likelihood of a positive anti‐ENA decreased. No other study examining multiple markers over individual markers of suspected AD patients has demonstrated the significance of C3 in diagnosis. Importantly, C3 was specifically associated with anti‐ENA rather than C3 and C4. With the complexity of AD diagnostic tools, specifying this complement toward anti‐ENA helps to simplify and define its importance in AD's presence.

High globulin levels can reflect increased globulin production, potentially resulting from increased humoral immune activity and/or increased inflammation, which are common in many ADs, aligning with our findings [24]. For example, increased globulin levels can be seen in active inflammatory bowel disease (IBD), rheumatoid arthritis (RA), and patients with Sjögren syndrome (SS); [25, 26, 27]. We demonstrated an increased probability of approximately 143% for having a positive anti‐ENA from increased globulin, aligning with the importance of globulin levels and ADs. Globulin is a component of the CMP commonly ordered by providers. With the significant increase in the odds of anti‐ENA in patient serum with the presence of globulin, it is important to not overlook this analyte among other testing within this panel.

Other than globulin, one of our most significant findings relates to the laboratory marker and ratio calculation, MLR. MLR has shown an association with ADs, with some usefulness in assessing disease severity [28]. Specifically, MLR has shown significant increases in patients with systemic autoimmune rheumatic diseases (SARDs; [29]). Higher monocyte counts or increased activity of various phenotypes can be seen in SLE, systemic sclerosis (SSc), RA, multiple sclerosis, type 1 diabetes, primary biliary cholangitis, SS, celiac disease, and IBD [30]. Lymphopenia has been observed in RA, insulin‐dependent diabetes mellitus, Crohn's disease, SLE, primary vasculitis, and pSS [31]. Within SLE, RA, and pSS, anomalies of lymphocyte subset distributions are common [32]. Idiopathic CD4 lymphopenia patients demonstrate a high prevalence of autoantibodies [33]. These findings contribute to the variance between increases in monocytes and decreases in lymphocytes, which results in increased MLR. In our study, increases in MLR improve the odds of a positive anti‐ENA by approximately 525%. With such a large increase in the odds of a positive anti‐ENA, MLR should be considered for use in patient assessment. This is particularly essential, as a CBC is a commonly ordered laboratory test that provides both monocyte and lymphocyte counts, which are needed to calculate the ratio. It is important to note that, generally, a CBC provides the absolute cell counts for these types of white blood cells but does not provide a calculated ratio. With a high percentage increase in the possibility of a positive anti‐ENA in relation to increases in MLR, it may be useful to begin including this ratio regularly or as a reflex calculation for healthcare providers rather than relying on high or low flags tagged to monocytes or lymphocytes alone. Although MLR has shown an association with ADs, multimarker studies are limited in demonstrating this. Specifically, Seringec Akkececi et al. [28] found MLR as a marker of remission for active disease, but this was only related to Takayasu arteritis [28]. We have indicated the potential for multiple ADs under anti‐ENA presence to show an association with MLR.

The final marker within our model, BUN, relates to a decrease in renal function, which can lead to an increase in urea nitrogen in the blood [14]. Krishnamurthy et al. [14] demonstrated higher mean BUN values in both male and female SLE subjects with a positive anti‐dsDNA compared to controls. Additionally, patients were tested for anti‐Sjogren's syndrome A (SSA Ro), anti‐Sjogren's syndrome B (SSB La), RNP/Sm, Polymyositis antibodies (Jo‐1), Sm, Scleroderma antibodies (Scl‐70), chromatin, centromere, histone, and RNA polymerase III within an anti‐ENA panel. Positive results for SSA Ro, SSB La, RNP/Sm, and Jo‐1 demonstrated a connective tissue disorder with higher mean values of BUN than the control group [14]. RA patients demonstrated elevated BUN levels compared to controls [14]. With decreased BUN, we demonstrated a 3.0% decrease in the probability of having a positive anti‐ENA. Decreases in BUN can be found in a common CMP panel, lending clarity toward the absence of anti‐ENA in patients.

### 7.2 Anti‐dsDNA

According to a multiple logistic regression model, complement C3, complement C4, urine pH, urine protein, and NLR significantly correlated with a positive anti‐dsDNA result. Of important note is the use of anti‐dsDNA as a marker for SLE, as approximately 70% to 98% of patients test positive [34]. Therefore, as expected, many significant factors are associated with SLE.

Although C3 alone was found to be significant in anti‐ENA positive patients, both C3 and C4 were significant in association to anti‐dsDNA. This is important as complement‐related to SLE pathogenesis is well accepted [35]. Both serum C3 and C4 levels are generally lower in SLE [35]. Within our study, we demonstrated that decreases in complement C3 and complement C4 lowered the probability of having a positive anti‐dsDNA value, aligning with the concept of complement activation from ADs. Previous multiple marker studies to determine a suspected AD have shown that C3 and C4 assist in the detection of anti‐dsDNA, and potentially even more importantly, play an essential role in the treatment and diagnosis of SLE [36]. Although there is a clear risk of over‐reliance on singular AD testing results regarding SLE, our study shows that interpretations and decisions from complement measures can potentially be relied upon.

Regarding the next laboratory marker, depending on the mechanism of the disease and the specific organ systems that are impacted, urine pH can fluctuate in patients with an AD. An acidic pH is a general marker for a potential AD with renal involvement [37]. ADs, including SLE, are associated with distal renal tubular acidosis, which would cause alkaline urine [38, 39]. These observations led to a discrepant nature regarding urine pH results for ADs. Due to this tendency, pH as an indicator, whether acidic or alkaline, should be considered with caution, specifically related to SLE. Within our study, we demonstrated that an acidic urine pH decreased the probability of a positive anti‐dsDNA, favoring the potential for alkaline urine pH to be associated with ADs. Therefore, it may be more appropriate to consider an alkaline urine result specifying distal tubular involvement within the kidney over an acidic result being general toward kidney damage as a whole.

Another common marker of ADs and renal involvement we examined is urine protein [37]. In the absence of urine protein, there was a significant decrease in the possibility of a positive anti‐dsDNA. SLE patients with lupus or non‐lupus‐related kidney disease may demonstrate low‐level proteinuria [40]. Current laboratory markers for lupus nephritis include proteinuria [35]. It is an essential screening test, as proteinuria demonstrating kidney involvement in SLE patients is one of the most typical manifestations [41]. Subsequently, a rapid urine reagent strip test is a quick method to analyze urine and proteinuria, potentially representing renal involvement as part of an AD. As the protein in urine is abnormal, proteinuria can be an inexpensive combined marker for AD diagnosis. If an AD diagnosis is established, it is critically important to frequently perform urinalysis for monitoring renal function [6]. In the case of an AD, it is also necessary to quantitatively determine proteinuria through current methods available such as 24‐h proteinuria or urine protein‐creatinine ratio. Based on urinalysis findings, including proteinuria and urine sediment, a kidney biopsy is indicated, as well as a therapeutic modality. Urine reagent strips can still provide a semiquantitative means for monitoring not only through point‐of‐care testing but also potentially through at‐home, self‐administered screening for proteinuria, saving time and resources and limiting healthcare spending. This may allow for a more efficient prognosis for patients who suffer from organ involvement within active SLE. This could be particularly important for patients with early lupus nephritis by self‐monitoring and seeking care when needed. Patients diagnosed with lupus nephritis still must have more frequent nephrological controls and quantitative determination of proteinuria in order to correct immunosuppressive therapy and monitor the course of the disease.

Progressing to potentially our most significant finding in association with the odds of a positive anti‐dsDNA occurring was NLR. NLR is widely used as a marker of immune response to infectious and noninfectious stimuli, along with a novel use as a marker of inflammation [42]. Previous research has also shown that increased NLR is useful to monitor disease activity and inflammation caused by SLE [28, 43]. Our study highlights the importance of blood cell ratios, specifically NLR's association to a positive anti‐dsDNA, further supporting the frequent pattern seen in studies of elevated neutrophil counts and lowered lymphocyte counts compared to healthy individuals [44]. Our study demonstrates that increased NLR is associated with a heightened probability of a positive anti‐dsDNA, which aligns with previous research on its elevation in SLE patients. Although previous studies have shown an association between NLR and SLE, our study strengthens this association using multiple markers, which is lacking in past research. NLR can be quickly calculated from the neutrophil and lymphocyte count provided within the CBC, allowing providers to add another useful measurement in SLE diagnosis. As discussed previously, regarding MLR and positive anti‐ENA patients, it may be useful to begin including this completed calculation with the CBC results, rather than relying on the absolute cell values and the LIS flagging the result as low or high.

## 8 Limitations

Some limitations should be considered while reviewing the data. As a retrospective study performed on de‐identified laboratory results, we cannot completely control for the potential of comorbid conditions that might have impacted results. Therapeutic treatment was not disclosed to the researchers, limiting the knowledge of the potential influence on laboratory results. Although the importance of ANA titer and pattern is known, we only included qualitative results. As discussed, anti‐ENAs have considerable overlap in associations with various ADs, and our study considered a general positive anti‐ENA result for the dependent variable instead of specifically analyzing singular analytes within the anti‐ENA panel.

### 8.1 Implications and Future Direction

Our research implies the potential use of specific diagnostic analytes to predict ADs not only through their presence or absence but also through the value of their results. Along with physical examination, healthcare providers should consider the use of general laboratory assay results that demonstrated significance in this study before considering the implementation of more expensive confirmatory testing. Specific attention should be paid to the presence of high globulin and MLR, as providers should begin to suspect the possibility of a positive anti‐ENA. Providers may utilize an elevated NLR as an indication regarding the possibility of an anti‐dsDNA. Future research should consider assessing similar laboratory tests and their association with specific extractable nuclear antigens. This application could help identify if the analytes included in our study are more specific to a particular AD. Several results on analytes that exhibited associations with ADs were not reproducible in this study. Further research is warranted to reproduce results showing which analytes included in the independent variables are significantly associated with anti‐ENA and anti‐dsDNA.

## 9 Conclusion

In conclusion, to the best of our knowledge, this is the first retrospective study to assess the full range of both essential general and specific diagnostic tests available that relate to confirming ADs. The predictive value and clinical utility of laboratory analysis are apparent to assist with AD diagnosis and potentially improve patient outcomes.

## Conflicts of Interest

The authors declare no conflicts of interest.

## Data Availability

Research data are not shared.

## Appendix A Definition of Terms and Acronyms

Autoimmune disease (AD): Elements of an organism's own immune system attacking tissue or cells by the formation of autoantibodies.

*Independent variables*

- Age (years)
- Sex (male, female)
- Comprehensive Metabolic Panel (CMP)
  ○ Albumin Globulin (AG) Ratio
  ○ Albumin
  ○ Alkaline Phosphatase
  ○ Alanine Aminotransferase (ALT)
  ○ Aspartate Aminotransferase (AST)
  ○ Bilirubin Serum
  ○ Blood Urea Nitrogen (BUN)
  ○ BUN Creatinine Ratio
  ○ Calcium
  ○ Chloride
  ○ Globulin
  ○ Creatinine
  ○ Potassium
  ○ Protein
  ○ Sodium
- C‐Reactive Protein (CRP)
- Complete Blood Count (CBC)
  ○ Eosinophil Absolute
  ○ Hematocrit
  ○ Lymphocytes Absolute
  ○ Monocytes Absolute
  ○ Neutrophils Absolute
  ○ Basophils Absolute
  ○ Platelets
  ○ Red Blood Cells (RBCs)
  ○ Red Blood Cell Width (RDW)
  ○ White Blood Cells (WBC)
  ○ Eosinophil‐to‐Lymphocyte Ratio (ELR)
  ○ Monocyte‐to‐Lymphocyte Ratio (MLR)
  ○ Neutrophil‐to‐Lymphocyte Ratio (NLR)
  ○ Platelet‐to‐Lymphocyte Ratio (PLR)
- Urinalysis (UA)
  ○ Occult Blood
  ○ pH
  ○ Protein Urine
  ○ Specific Gravity
  ○ WBC Esterase
  ○ Bilirubin Urine
- Erythrosedimentation Rate (ESR)
- Anti‐Nuclear Antibody (ANA Screen)
- Complement C3
- Complement C4

*Dependent variables*

- Anti‐Extractable Nuclear Antigen (Anti‐ENA) Screen
- Anti‐Double Stranded DNA (Anti‐dsDNA) Screen

*Operational definitions*

Anti‐ENA and anti‐dsDNA by multiplex immunoassay determined the levels of autoantibodies in serum. Indirect Immunofluorescence (IFF) assay determined serum present ANAs. CRP values were collected through latex immunoturbidimetry methodology. CBC results were recorded through an automated cell counter with mixed technologies. CMP results were collected through mixed technologies. Urinalysis results were collected through a reagent strip. Automated and manual modified Westergren methods were employed for ESR. C3 and C4 were analyzed through turbidimetry.
